# Individual risk factors and prediction of gambling disorder in online sports bettors - the longitudinal RIGAB study

**DOI:** 10.3389/fpsyt.2024.1320592

**Published:** 2024-02-27

**Authors:** Theresa Wirkus, Robert Czernecka, Gerhard Bühringer, Anja Kräplin

**Affiliations:** ^1^Department of Psychology, Institute for Clinical Psychology and Psychotherapy, Technische Universität Dresden, Dresden, Germany; ^2^Institut für Therapieforschung IFT, Prävention und betriebliche Gesundheitsförderung GmbH, München, Germany; ^3^Department of Clinical Research, Faculty of Health, University of Southern Denmark, Odense, Denmark

**Keywords:** gambling disorder, online sports betting, online gambling, individual risk factors, longitudinal study

## Abstract

**Introduction:**

While research in online sports betting is dominated by studies using objective player tracking data from providers to identify risky gambling behavior, basicresearch has identified various putative individual risk factors assumed to underlie the development of gambling disorder across all types of gambling. This study aims to examine individual risk factors and their longitudinal clinical relevance in online sports bettors.

**Methods:**

German online sports bettors (*N* = 607, *M_age_* = 34, 92% male) from a provider based sample took part in an online survey. The study team randomly preselected customers to be invited. *N* = 325 (53,45%) of the participants also took part in an online follow-up survey one year later. Crosssectional and longitudinal associations of putative risk factors and DSM-5 gambling disorder in online sports bettors were analyzed. These risk factors include alcohol and tobacco use, impulsivity, difficulties in emotion identification, emotion regulation strategies, comorbid mental disorders and stress.

**Results:**

We found more pronounced impulsivity, difficulties in emotion identification, emotion suppression, comorbid mental disorders and stress were cross-sectionally associated with gambling disorder, and longitudinally predicted gambling disorder in online sports bettors (with the exception of emotion suppression). In an overall model only lack of premeditation and perceived helplessness remained significant as predictors for gambling disorder. Online sports bettors with gambling disorder predominantly showed more pronounced risk factors, which were also confirmed longitudinally as relevant for the maintenance of gambling disorder.

**Discussion:**

Risk factors such as impulsivity and stress and appropriate coping mechanisms should consequently be integrated not only into prevention efforts to identify individuals at risk early, but also into intervention efforts to tailor treatment.

## 1 Introduction

More and more people are engaging in online gambling (1, 2) such as online sports betting. In Germany, where the present study took place, this is for instance mirrored in an increasing turnover and market share of online sports betting (3). The main objectives of this paper are 1) to characterize online sports bettors concerning selected putative individual risk factors for gambling disorder (GD), which have been established in studies on gambling in general (4) and 2) to further use this risk profile to predict GD after one year.

Disordered gambling with past-year prevalences ranging between .12% – 5.8% worldwide (5) and .34% - 2.3% in Germany in particular (6, 7) incurs considerable costs for individuals and society (8). A variety of terms, e.g. “problem gambling”, are employed to describe risky gambling behavior resulting in a range of adverse consequences for the individual or its environment (9) without specifying its clinical significance. In the present study we focused on clinically significant behavior based on the current criteria for GD from the Diagnostic and Statistical Manual of Mental Disorders (DSM-5, 5^th^ edition, 10). Within this article, when describing other studies, we will however employ the terms used by the respective authors.

At the time of our study, online sports betting was among the only at least partly legalized forms of online gambling in Germany, which is why we focused on this form of gambling. In July 2021, a new legislation (11) came into force in Germany legalizing online gambling in the form of lotteries, sports betting, horse racing, casino, and poker. Prior to that, from 2012 to July 2021, the regulation of online gambling was rather complex; as it was legal in only one of the 16 German Federal States. In all other states online gambling had no legal basis due to public health concerns, but there was no prosecution.

Accessibility, availability, privacy and anonymity are among the factors being discussed in relation to online gambling posing more risk for harm than offline gambling (e.g. 12). Furthermore, several studies and a meta-analysis found online gambling to be associated with a higher likelihood of problem gambling compared to offline gambling (4, 13–15). However, these studies have among other things been criticised for the attempt to measure the “pure” effect of online gambling, accompanied by heterogeneous operationalisations of the distinction between online and offline gamblers (e.g. 16). In this context several studies found mixed-mode gamblers to exhibit the highest level of gambling problems (17–19), presumably because people experiencing problems are more involved in gambling in general (20), i.e. they gamble in various ways and modes. While the potential causal influence of online gambling in respect to gambling problems remains unclear (e.g. 16), its relevance with regard to a constantly growing online gambling market within the EU and sports betting as one of its most popular products (21) is indisputable. This in turn makes it necessary to understand the respective risk factors and also develop better prevention measures for GD in online gambling. In this regard two research approaches seem relevant.

First, research in online gambling is dominated by studies using objective player tracking data from provider based samples, aiming to inform prevention measures such as early detection of players at risk (e.g. 22–24). In these studies risky gambling was often conceptualized through account based proxies such as account closure (e.g. via self-exclusion); an approach which has been criticized for limitations like the lack of evidence for a direct relationship with problem gambling (25).

A different, clinically more valid approach has been taken by a few studies, which used player tracking data from provider based samples to predict clinically validated screenings of GD (26–30). Most of them used the Problem Gambling Severity Index (PGSI, 31) or the brief biosocial gambling screen (BBGS, 32) as outcomes. To the best of our knowledge, none of them explored other individual risk factors supposed to be relevant in the development and course of GD.

Second, regarding the development of GD in general various factors are discussed within etiological research (see 4 for a meta-analysis; see 33 for a systematic review). Within this paper, we want to focus on selected putative risk factors for GD that have already been established across different types of gambling. These factors include among others dysfunctional emotion regulation processes (34, 35), various comorbid mental disorders, with the most prevalent being substance use and respective disorders, affective and anxiety disorders (36) and heightened impulsivity (37, 38).

There is evidence for a cross-sectional association of some of these factors like substance use, other aspects of mental health and high impulsivity with problem gambling in online gamblers (16, 20) and in sports bettors in general (39–41; see 42 for a review). However, to the best of our knowledge these putative risk factors have not been analyzed in online sports bettors in particular yet.

To address this research need and taking into account existing risk profiles of gamblers with GD (i.e. high impulsivity, increased tobacco and alcohol use, difficulties in emotion identification, dysfunctional emotion regulation strategies, increased comorbid mental disorders and stress), we now characterize online sports bettors with and without GD according to DSM-5 concerning these established risk factors for the first time. Furthermore, we aimed to examine the individual risk factors from a less researched longitudinal perspective, to add to the literature on their potential causal role in the entire course, that is to say the potential onset, recurrence, remission and maintenance of GD.

We hypothesize that online sports bettors with GD have more pronounced individual risk factors than those without GD and that these risk factors have a predictive value for GD one year later [see preregistrations for details https://osf.io/jbfhe, https://osf.io/5qxmh (43, 44)]. In this regard, we were interested in exploring the relevance of the respective single putative individual risk factors in order to provide starting-points not only for future investigations but also prevention and intervention efforts. Please note that within this manuscript, we use ‘predict’ in a technical sense to indicate a relationship between the ‘predictor’ variables and outcomes of the logistic regressions, and not to suggest these predictor variables cause GD.

With our approach, we aim to bridge the gap between etiological and preventive research in online sports betting and to provide information for improved prevention and intervention programs adapted to the specific characteristics of online sports bettors with GD, which may also be useful for other types of online gambling.

## 2 Materials and methods

We preregistered hypotheses and analysis plans for each study part separately on the Open Science Framework [initial online survey https://osf.io/jbfhe, follow-up online survey https://osf.io/5qxmh (43, 44)]. This is the first of multiple planned publications concerned with results from the RIGAB study. The current paper addresses hypotheses 1–5 of the preregistration for the initial online survey and hypotheses 5-11 of the preregistration for the follow-up online survey.

### 2.1 Participants and recruitment

For initial recruitment, we cooperated with Tipico, an international gambling provider. Before recruitment, Tipico provided us with anonymous player tracking data for all German customers. The anonymous player tracking data consisted exclusively of online sports betting data, usually placed via private devices (e.g. smartphone, tablet). Our study group then included customers meeting the following criteria in the sample: aged between 18 and 55 years, having logged into their account in the two months prior to the beginning of the study and with a current age of their account of minimum 6 months. As a later part of the RIGAB study was conducted in-person, another inclusion criterion was living close to one of the study locations in Germany (Dresden, Leipzig, Chemnitz, Munich, Berlin, Hamburg, Frankfurt am Main, Düsseldorf). Different from the preregistration we had to add the last two locations later, since the response rate in the other cities was not sufficient to reach the planned sample size (45).

At the time sampling was started, the prevalence for GD in Germany was estimated rather low at .34 -.82% (2009-2019) (6). Since we wanted to reach a sufficient number of players with GD, we made use of an artificial intelligence (AI) used within the provider’s responsible gambling strategy, which classified the players into a group with and a group without risky gambling behavior. The AI’s algorithm is a self-learning successive individual risk evaluation, which does not screen for GD but for previously defined indicators that could predict “gambling related problems” in player tracking and player communication data and evaluates them over time (46). It is based among other things on age and exposure, but also on behavioral parameters like the number of games played and gambling days derived from player tracking data studies also mentioned above (e.g. 26), complaint behavior, or observation of strong emotions (in a Tipico shop, at the hotline, via e-mail) but also other not (publicly) defined indicators, which have been proven valid for risk categorization with the provider and the player’s betting history (46). According to Tipico, the algorithm shows good diagnostic properties to indicate gambling related problems. This classification was used for recruitment purposes only.

As we assumed the response rate would be lower for players with risky gambling behavior, we randomly selected these customers to be invited to participate in our study first. Next, we randomly selected customers without risky gambling behavior to be invited. Contrary to the preregistration, we did not match persons of the respective groups on selected control variables. Since the recruitment of the group with risky gambling behavior took longer than planned, we had to start inviting the group without risky gambling behavior before recruitment in the group with risky gambling behavior was completed and thus before the specific characteristics of the matching variables for the final sample were known. To account for this, we included (as preregistered) all proposed matching measures as control variables in all our analyses. After we had randomly selected customers to be invited to participate, the provider assisted us by sending out invitations in waves from May to July 2021 due to data protection reasons. A total of *n* = 3268 players with risky gambling behavior and *n* = 3300 players without risky gambling behavior were invited. Of the players with risky gambling behavior, *n* = 2668 were invited in the first three waves, consisting of two waves with 1000 customers and one with 668 customers. These waves consisted of all players with risky gambling behavior who met the original inclusion criteria. From the response rates of these waves, we deduced how many players we needed to invite from each group for the final wave. In the fourth and last wave, 600 customers with risky gambling behavior from the resampled study locations were invited together with 3300 customers without risky gambling behavior. Please see the preregistration of the initial online survey (https://osf.io/jbfhe) or the study protocol (45 for further details on the recruitment process.

**Figure 1:**
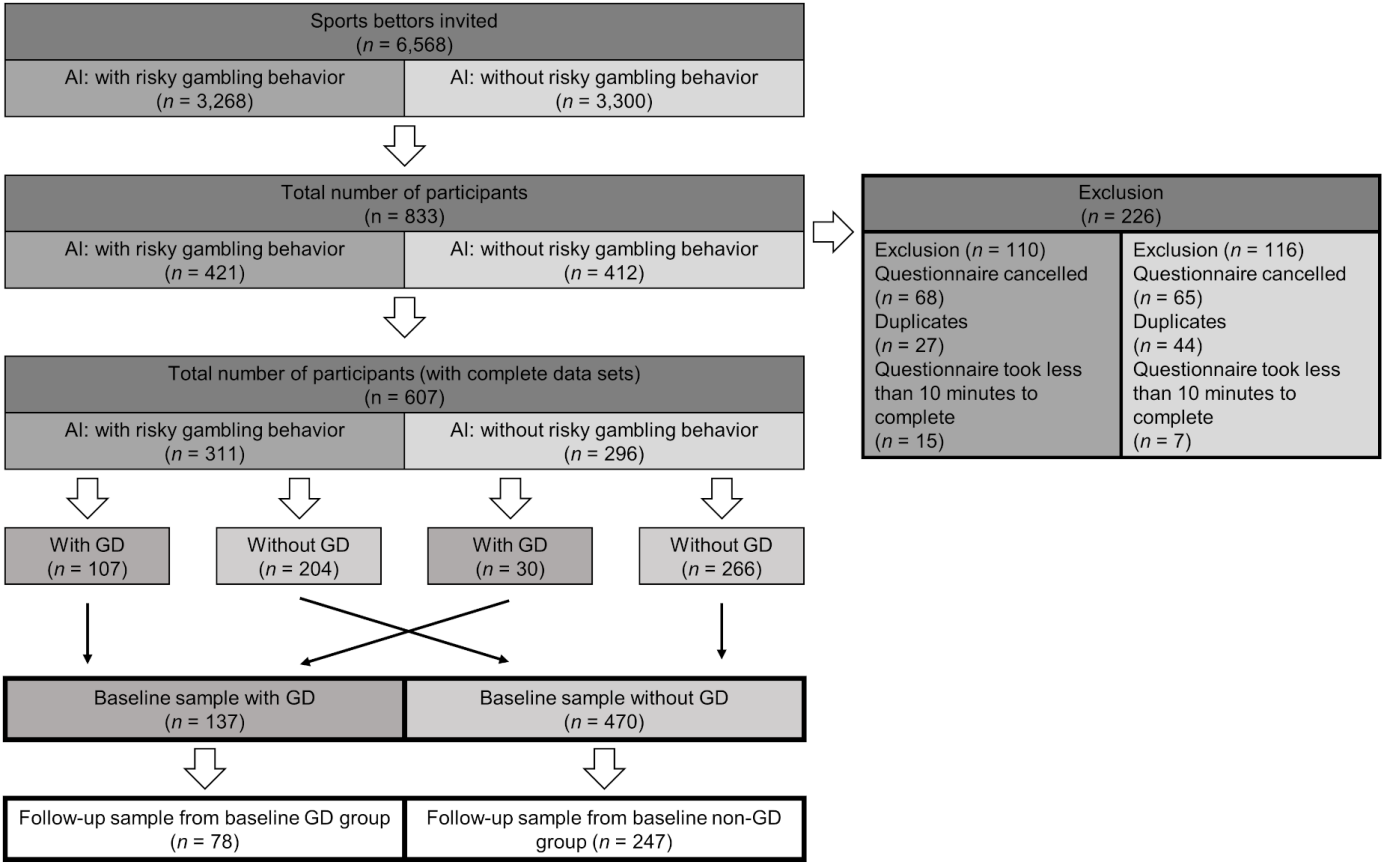
Flow chart of participants of the RIGAB study (47). AI, Artificial Intelligence, classification according to provider’s algorithm. GD, Gambling Disorder assessed with DSM-5 Stinchfield screening questionnaire.

**Figure 1** depicts a flowchart of the participants of the initial online survey. One year after the initial online survey we invited all participants, who had agreed to be contacted for further study parts, to participate in the follow-up online survey.

### 2.2 Measures

#### 2.2.1 GD

We screened for GD with an internal German translation of the DSM-5 Stinchfield criteria concerning the last 12 months (adapted from 48). DSM-5 criteria have demonstrated satisfactory reliability, validity and classification accuracy (49). Although various DSM-5 based screenings have been used in multiple epidemiological studies in Germany (e.g. 50), there are no German validation studies. For this reason, Cronbach’s alpha presented here was calculated with our own data. In the current study, Cronbach’s alpha was .84 for the initial and .87 for the follow-up online survey. Participants were asked about all nine diagnostic criteria for GD according to DSM-5 on a dichotomous scale. Corresponding to DSM-5 the clinical cut-off for a diagnosis is four fulfilled criteria.

#### 2.2.2 Alcohol and tobacco use

Measures used to assess alcohol and tobacco use were taken from the German version of the WHO Composite International Diagnostic Interview (DIA-X/M-CIDI, 51). In the following, we use the questions concerning tobacco use for illustration purposes. The questions concerning alcohol use were similar. Respective substance use was measured by asking participants about the frequency of use (e.g. “How often did you smoke cigarettes or something comparable in the past 12 months?”), where possible answers (never – less than once per month – more than once per month: e.g. 5 days per week) were transformed to indicate consumption occasions per week. Furthermore, participants were asked about the quantity of their use of alcohol and tobacco for the past 12 months (e.g. “During a typical week, in which you smoke, how many cigarettes or something comparable do you smoke per day?”) and their customary form of consuming it (e.g. cigarettes, vapes etc.). With this information, we computed a modified version of the quantity-frequency-index (QFI, 52) by multiplying the average consumption per occasion (e.g. 10 cigarettes) by the number of average weekly consumption occasions (e.g. 7 days per week), indicating the average consumption per week.

#### 2.2.3 Impulsivity

Impulsivity was measured with a short version of a German version of the UPPS-P (earlier version of 53), which has yielded good psychometric properties. Participants indicate their level of general agreement to impulsivity related statements on a 4-point Likert-scale. After appropriate reverse coding, the respective sum scores for the five subscales positive urgency, negative urgency, sensation seeking, lack of premeditation and lack of perseverance were computed. Since we used an earlier partly different version of the measure with no published information on psychometric properties, Cronbach’s alpha presented here was calculated with our own data. Cronbach’s alpha in our study ranged between .77 (sensation seeking) and .86 (positive urgency).

#### 2.2.4 Difficulties in emotion identification and emotion regulation strategies

Difficulties in emotion identification were measured with the sum score of the respective subscale of the German version of the Toronto Alexithymia Scale (TAS-26, 54). Emotion regulation strategies were measured with the German version of the Emotion Regulation Questionnaire (ERQ, 55). Both measures have yielded good psychometric properties. Participants rated how much they agreed with certain statements about themselves in general on a 5- (TAS-26) and 7-point (ERQ) Likert-scale respectively. For analysis the sum score of the TAS-26 subscale was computed. For the ERQ subscales, reappraisal and suppression respective mean scores were computed after appropriate reverse coding.

#### 2.2.5 Comorbid mental disorders

Comorbid mental disorders were measured with a German version of the Brief Symptom Inventory (BSI-18, 56), which has yielded satisfactory to good psychometric properties. Participants stated whether they experienced selected symptoms of depression, anxiety and somatization in the last 7 days on a 4-point Likert-scale. The sum score of all items of the respective subscales, which is also called global severity index (GSI) and respective subscales were computed for analyses.

#### 2.2.6 Stress

Perceived stress was measured with a German version of the Perceived Stress Scale (PSS-10, 57), which has yielded very good psychometric properties. Participants stated how frequently they perceived stress in the last month on a 5-point Likert scale. Sum scores for both subscales perceived helplessness and perceived self-efficacy were calculated. For the total sum score, self-efficacy items were reversed.

In both online surveys participants also filled out information on sociodemographics, gambling behavior, motives etc., which will not be discussed here [see https://osf.io/ac8gj for full account of all used instruments within the study (58)].

### 2.3 Procedure

The initial online survey is the first part of the longitudinal RIGAB study (for more details see preregistrations https://osf.io/k6c23/ (59) or 45). Data for both online surveys were collected using the secure, web-based software platform Research Electronic Data Capture (REDCap, 60, 61) hosted at Technische Universität Dresden. After providing informed consent, the participants were asked to fill out the aforementioned questionnaires in the initial survey. The follow-up survey included only selected questionnaires including a screening for GD [see https://osf.io/5qxmh for full account of all used instruments within the follow-up study (44)].

All participants filled out the same survey in the same order, which took about 30 minutes for the initial and 10 minutes for the follow-up online survey. Participants received an amazon voucher of 30 respectively 10 euros as compensation. After completion of the initial online survey participants could decide, whether or not they wanted to provide their contact details to be invited to participate in further study parts.

### 2.4 Statistical analysis

All analyses were conducted with the STATA 14.2 software package (62). Our first objective was to examine the risk profile of online sports bettors with and without GD (binary, independent variable) concerning selected putative individual risk factors (dependent variable) at the initial online survey. For this, bettors were classified as with (≥4 fulfilled criteria) or without GD according to the number of fulfilled DSM-5 criteria, this variable was dummy-coded. In this cross-sectional analysis we calculated separate multiple linear regressions for each putative individual risk factor as outcomes, using the dummy coded GD group as independent variable.

Our second objective was to examine whether players with more pronounced risk factors at the initial online survey (independent variable) have a heightened probability of GD one year later (binary, dependent variable). For this longitudinal analysis, we calculated logistic regressions separately for each putative individual risk factor, this time using the dummy coded GD diagnosis as outcome.

All analyses (multiple and logistic regressions) were conducted separately for each putative individual risk factor as we were interested in the respective relevance of these single factors, because we consider them future starting-points for both continuing investigations and treatment and prevention efforts.

Further exploratory analyses were added to our preregistered analyses, as interest in these emerged during the review process. Based on a reviewer’s comment, we also explored the relative predictive relevance of all individual risk factors for a GD diagnosis one year later. For this longitudinal analysis, we conducted a logistic regression including all individual risk factors examined here as independent variables in an overall model. The dummy-coded GD diagnosis at follow-up was used as the outcome (binary, dependent variable) again.

Based on previous research in the field, which has identified several sociodemographic factors as putative risk factors for GD (63, 64), we included age, gender, education and age of betting account as a proxy for online gambling exposure (23) as covariates in all analyses (both multiple and logistic regressions).

To determine whether the study retention group of our follow-up sample differed significantly from the drop-out concerning covariates or independent variables, we conducted Chi2-, t- or Mann-Whitney-U-tests depending on the distribution and level of measurement of the respective variables.

Based on a reviewer’s comment, we also present explorative analyses testing whether participants differ in the individual risk factors examined here depending on the course of GD over one year. To examine whether putative risk factors play different roles during the course of GD, we compared participants showing stable GD, onset, or remission, over one year with the group of participants who had no GD at both time-points (stable non-GD).

### 2.5 Ethics

The study procedures were carried out in accordance with the Declaration of Helsinki. The Institutional Review Board (IRB00001473) of Technische Universität Dresden approved the study protocol under the reference SR-EK-190032021. Before beginning the initial online survey, all subjects were informed about the study and all subjects provided informed consent.

## 3 Results

Descriptive characteristics of the initial sample are shown in **Table 1**. Participants were predominantly male (92%) with an average age of 34 years. The majority had a high level of education (*n* = 334 (55%)) and an average age of account of 5.45 (2.98) years. Of the initial sample *n* = 137 were classified as GD and *n* = 470 as no GD according to our DSM-5 screening. When asked how frequently they participated in 16 common forms of gambling in Germany besides online sports betting with the provider, most particpants across all groups indicated their most frequently played form of gambling besides online sports betting with Tipico was online sports betting with other providers (e.g. *n* = 278 (45.8%) in the overall sample).

**Table 1:** Descriptive characterization of the initial online survey sample.

	All	GD	No GD
Age, *M (SD)*	34 (8.85)	33 (7.61)	34 (9.17)
Gender (male)	*n* = 557 (92%)	*n* = 125 (92%)	*n* = 432 (92%)
Education			
low	*n* = 58 (10%)	*n* = 18 (13%)	*n* = 40 (9%)
middle	*n* = 204 (34%)	*n* = 50 (37%)	*n* = 154 (33%)
high	*n* = 334 (55%)	*n* = 64 (47%)	*n* = 270 (57%)
other	*n* = 11 (2%)	*n* = 5 (4%)	*n* = 6 (1%)
Sum of DSM-5 Stinchfield criteria, *M (SD)*	2.09 (2.37)	5.93 (1.56)	.97 (.99)
Age of betting account, *M (SD)*	5.45 years (2.98)	5.69 years (3.17)	5.38 years (2.92)
German as a native language	*n* = 508 (84%)	*n* = 92 (67%)	*n* = 416 (89%)
Most common family status	*n* = 414 single (68%)	*n* = 97 single (71%)	*n* = 317 single (67%)
Most common employment status	*n* = 449 (74%) fully employed	*n* = 91 (66%) fully employed	*n* = 358 (76%) fully employed
Age at first gamble, *M (SD)*	20.36 (6.1)	19.5 (5.32)	20.62 (6.23)
Top 5 most frequently played forms of gambling besides online sports betting with the provider			
1	Online sports betting (other providers) *n* = 278 (45.8%)	Online sports betting (other providers) *n* = 74 (54.01%)	Online sports betting (other providers) *n* = 204 (43.4%)
2	Sports betting facilities *n* = 109 (17.96%)	Sports betting facilities *n* = 38 (27.74%)	Lottery *n* = 80 (17.02%)
3	Lottery *n* = 99 (16.31%)	Other online gambling *n* = 31 (22.63%)	Sports betting facilities *n* = 71 (15.11%)
4	Other online gambling *n* = 72 (11.86%)	Lottery *n* = 19 (13.87%)	Other online gambling *n* = 41 (8.72%)
5	Gambling on stock market *n* = 56 (9.23%)	Gaming machines *n* = 19 (13.87%)	Gambling on stock market *n* = 41 (8.72%)

*N_total sample_* = 607, *n_GD_* = 137, *n_no GD_* = 470. *M*, Mean; *SD*, Standarddeviation. Where applicable means and standarddeviation are displayed, for non-metric variables absolute and relative frequencies in percent are displayed. Participants were asked how frequently they played 16 common forms of gambling in Germany besides online sports betting with the provider on a 5-point scale (never – very often), absolute and relative frequencies displayed indicate the shares of the respective groups, who replied “often” or “very often”.

From the initial online survey *n* = 325 (retention rate 53,45%) participated in the follow-up online survey. Participants of the follow-up survey were predominantly male (94%) with an average age of 35 years and an average age of account of 5.47 (2.96) years. The average number of fulfilled DSM-5 Stinchfield criteria for GD at follow-up was 2.12 (range 0-9) with *n* = 75 classifying as GD and *n* = 250 as no GD according to our DSM-5 screening.

In the following, only a few selected results are given as examples below. Due to the extensive amount of results, all other results and means of the multiple regressions testing whether online sports bettors with GD at the initial online survey have more pronounced putative risk factors than those without GD are shown in **Table 2**. Amongst others, we found higher indicators for impulsivity like positive (*b* = 1.46, [.93, 1.99]) and negative urgency (*b* = 1.8, [1.26, 2.34]), more difficulties in emotion identification (*b* = 3.72, [2.54, 4.9]), higher indicators for comorbid mental disorders like depression (*b* = 3.69, [2.93, 4.44], and for stress (*b_PSS-10 sum score_* = 5.96, [4.88, 7.05]) in bettors with GD.

**Table 2:** Results of the multiple linear regression analyses for cross-sectional differences in putative individual risk factors between sports bettors with and without gambling disorder at the initial online survey.

Outcome	*Mean_GD_ (SD)*	*Mean_no GD_ (SD)*	Regression coefficient	*p*	95% CI
Substance use					
QFI alcohol	39.55 (77.54)	57.72 (106.17)	-13.89	.16	[-33.28, 5.5]
QFI tobacco	44.31 (61.92) (*n* = 137)	33.18 (53.45) (*n* = 468)	7.27	.168	[-3.062, 17.61]
Impulsivity					
Sensation seeking	9.02 (3.59)	9.27 (3.56)	-2.63	.437	[-.93,.4]
Lack of premeditation	9.17 (2.71)	7.67 (2.43)	1.4	<.001	[.91, 1.89]
Lack of perseverance	8.16 (2.63)	7.01 (2.47)	1.07	<.001	[.58, 1.55]
Positive urgency	8.42 (2.62)	6.89 (2.81)	1.46	<.001	[.93, 1.99]
Negative urgency	10.07 (2.86)	8.17 (2.78)	1.8	<.001	[1.26, 2.34]
Difficulties in emotion identification and emotion regulation strategies					
Diff. emot. ident.	15.49 (7.22)	11.41 (5.66)	3.72	<.001	[2.54, 4.9]
Emot. reg. strateg.					
Suppression	4.11 (1.21)	3.68 (1.25)	.41	.001	[.17,.65]
Reappraisal	4.07 (1.12)	3.92 (1.1)	.14	.198	[-.07,.35]
Comorbid mental disorders					
General Severity Index	12.63 (13.16)	5.21 (6.91)	7.22	<.001	[5.53, 8.91]
Depression	5.59 (5.6)	1.9 (3.2)	3.69	<.001	[2.93, 4.44]
Anxiety	4.24 (4.72)	1.94 (2.43)	2.21	<.001	[1.61, 2.81]
Somatization	2.8 (4.25)	1.37 (2.47)	1.32	<.001	[.75, 1.9]
Stress					
PSS-10 Sum score	29.38 (5.66)	23.2 (5.56)	5.96	<.001	[4.88, 7.05]
Perceived helplessness	17.84 (4.84)	12.96 (4.26)	4.83	<.001	[3.98, 5.68]
Perceived self-efficacy	12.46 (2.76)	13.76 (3.14)	-1.13	<.001	[-1.73, -.54]

If not otherwise indicated *N_total sample_* = 607, *n_GD_* = 137, *n_no GD_* = 470. *SD*, Standarddeviation; CI, 95% Confidence Interval; QFI, quantity-frequency-index. Multiple regressions were conducted for each putative individual risk factor separately. All analyses controlled for the influence of age, gender, education and age of account.

In the following, only a few selected results are given as examples below. Due to the extensive amount of results, all other results of the logistic regressions testing whether online sports bettors with more pronounced putative risk factors at the initial online survey have a heightened probability of GD one year later are shown in **Table 3**. In sum, we found an increased risk for GD one year later in bettors with higher indicators of impulsivity except for sensation seeking, more difficulties in emotion identification and more pronounced indicators of comorbid mental disorders and perceived stress. For instance we found that, holding all control variables constant, the odds of a GD diagnosis increased by 36% [1.21, 1.52] for a one-unit increase in lack of premeditation and by 19% [1.12, 1.28] for a one-unit increase in depression.

**Table 3:** Results of the logistic regressions (longitudinal analysis) testing whether putative individual risk factors predict gambling disorder at follow-up.

	*OR*	*p*	95% CI
Substance use			
QFI alcohol	.995	.06	[.99, 1]
QFI tobacco	1.00	.301	[.998, 1.01]
Impulsivity			
Sensation seeking	.95	.23	[.88, 1.03]
Lack of premeditation	1.36	<.001	[1.21, 1.52]
Lack of perseverance	1.33	<.001	[1.19, 1.49]
Positive urgency	1.25	<.001	[1.13, 1.38]
Negative urgency	1.25	<.001	[1.13, 1.38]
Difficulties in emotion identification and Emotion regulation strategies			
Diff. emot. ident.	1.09	<.001	[1.05, 1.13]
Emot. reg. strateg.			
Suppression	1.22	.07	[.98, 1.52]
Reappraisal	1.05	.699	[.82, 1.34]
Comorbid mental disorders			
General Severity Index	1.08	<.001	[1.04, 1.11]
Depression	1.19	<.001	[1.12, 1.28]
Anxiety	1.19	<.001	[1.09, 1.28]
Somatization	1.12	.006	[1.03, 1.22]
Stress			
PSS-10 Sum score	1.17	<.001	[1.12, 1.23]
Perceived helplessness	1.22	<.001	[1.15, 1.3]
Perceived self-efficacy	1.19	<.001	[1.09, 1.31]

n = 325, OR, Odds Ratio; CI, 95% Confidence Interval; QFI, quantity-frequency-index. Logistic regressions were conducted for each putative individual risk factor separately. All analyses controlled for the influence of age, gender, education and age of account. Due to insufficient cell counts 2 categories of education had to be subsumed for analysis.

Results of the logistic regression including all individual risk factors at the initial online survey in an overall model and testing for their respective relevance in predicting GD one year later are shown in **Table 4**. We found that in an overall model only lack of premeditation with an OR = 1.26 (*p* = .006, [1.07, 1.48]) and perceived helplessness with an OR = 1.15 (*p* = .003, [1.05, 1.25]) remained significant predictors for a GD diagnosis at follow-up.

**Table 4:** Results of the logistic regression (longitudinal analysis) testing the relative predictive relevance of all putative individual risk factors in predicting gambling disorder at follow-up in an overall model.

	*OR*	*p*	95% CI
Substance use			
QFI alcohol	1.00	.099	[.99, 1]
QFI tobacco	1.00	.562	[.99, 1]
Impulsivity			
Sensation seeking	.94	.196	[.85, 1.03]
Lack of premeditation	1.26	.006	[1.07, 1.48]
Lack of perseverance	1.1	.222	[.94, 1.28]
Positive urgency	.98	.758	[.84, 1.14]
Negative urgency	1.07	.316	[.94, 1.23]
Difficulties in emotion identification and Emotion regulation strategies			
Diff. emot. ident.	1.03	.365	[.97, 1.09]
Emot. reg. strateg.			
Suppression	.91	.52	[.67, 1.21]
Reappraisal	1.15	.378	[.84, 1.57]
Comorbid mental disorders			
Depression	1.09	.162	[.97, 1.22]
Anxiety	1.01	.891	[.87, 1.17]
Somatization	.89	.159	[.75, 1.05]
Stress			
Perceived helplessness	1.15	.003	[1.05, 1.25]
Perceived self-efficacy	1.04	.526	[.91, 1.19]

n = 325; OR, Odds Ratio; CI, 95% Confidence Interval; QFI, quantity-frequency-index. The logistic regression was conducted with an overall model including all putative individual risk factors simultaneously. The analysis controlled for the influence of age, gender, education and age of account. Due to insufficient cell counts 2 categories of education had to be subsumed for analysis. The sum scores concerning comorbidities and stress had to be omitted due to collinearity.

Results of the dropout analyses for the follow-up survey are displayed in **Table S1** in the supplement. We found participants, who dropped out, tended to be younger (z = -2.65, *p* = .008) and have higher perceived self-efficacy (z = 2.2, *p* = .028). Though these differences were significant, they are only minimal in absolute terms concerning perceived self-efficacy. Concerning age, the reduced mean age in the drop-out group (*M*_drop-out_ = 33 (8.89)) points towards a possible selection bias, which is however acknowledged in our analyses since we controlled for the influence of age in all of them.

Results of the explorative analyses testing, whether participants differ in the individual risk factors examined here depending on the course of GD over one year (stable GD, onset, remission, stable non-GD) are displayed in the supplement (**Table S2**). Overall, we found significant differences in individual risk factors between individuals with stable GD and those without GD (stable non-GD) over one year. These distinctions remained stable over time, supporting their longitudinal relevance. Participants at different stages of GD (stable, onset, remission) exhibited significant differences from those without GD for certain risk factors. For some other factors, we found significant differences between those in remission and those without GD. However, limited sample sizes in the onset and remission groups may affect the generalizability of conclusions for these subgroups.

## 4 Discussion

The objectives of this paper were 1) to characterize online sports bettors concerning selected putative individual risk factors for GD and 2) to further use this risk profile to predict GD after one year. We replicated existing literature on more pronounced individual risk factors like heightened impulsivity and comorbid mental disorders in players with GD across different forms of (online) gambling specifically in online sports bettors 1). As the first longitudinal study, we could predict GD from the putative risk factors impulsivity, difficulties in emotion identification, comorbid mental disorders and stress and thus added an important temporal dimension to the existing literature 2).

In the following, cross-sectional and longitudinal findings for each putative risk factor will be discussed together to provide a more comprehensive interpretation of the results. In this section, we use ‘predict’ in a technical sense to indicate a relationship between ‘predictor’ variables and outcomes of the logistic regression, and not to suggest these predictor variables cause GD.

### 4.1 Impulsivity, emotion identification and emotion regulation

Consistent with our hypothesis, online sports bettors with GD demonstrated higher impulsivity than those without; except for sensation seeking, which adds to a number of heterogeneous results concerning this facet (65–67). Trait impulsivity has otherwise been consistently associated with GD in clinical and non-clinical samples (e.g. 66, 68). In our study, the various impulsivity facets show the strongest predictive value of all putative individual risk factors, increasing the likelihood of GD after one year by about 30%, supporting impulsivity as one of the core mechanisms underlying GD (69, 70). This is underlined by the fact that lack of premeditation was one of the two remaining significant predictors in the longitudinal overall analysis. This finding also suggests that impulsivity is related to other individual risk factors explored here as they seem to share explained variance. Considering that the latter seems to be true for almost all individual risk factors of the study, this might indicate an underlying more general and broader vulnerability concept encompassing several or all of these factors. The common liability to addiction theory describes such a general vulnerability, taking into account evidence from various studies with a neurological and genetics focus (71, 72). Using factor analysis, individual risk factors examined here could be explored along these lines in future studies.

(Negative) urgency, as a form of mood related impulsivity, is among the impulsivity facets with the strongest associations with GD (73–75). In our study, sports bettors with GD demonstrated the highest impulsivity related mean in negative urgency, exhibited more difficulties in emotion identification and were more likely to use suppression as a (maladaptive) way of emotion regulation. Difficulties in emotion identification were also found to be predictive of GD, which highlights the respective relevance as a putative risk factor and is consistent with previous cross-sectional studies (76–78). These findings further support a line of research arguing that gambling or addictive behaviors in general might be used as a (maladaptive) way to regulate emotions (e.g. 34), which has often been linked to a subtype of gamblers termed “emotionally vulnerable” (79, 80).

Taken together our data support the relevance of impulsivity for online sports bettors, consequently suggesting the necessity of its inclusion into prevention efforts, for example in the form of information about impulsive forms of gambling, i.e. encouraging bettors to research their bets, placing them in advance, and avoid live-action bets (40). Likewise impulsivity related interventions like goal-management training (81) along with identifying and regulating emotions for example with mindfulness based techniques should be integrated into treatment efforts.

### 4.2 Alcohol and tobacco use

We found no difference in alcohol or tobacco use between online sports bettors with and without GD. This is contrary to our hypothesis and to other studies in the field, which describe substance use disorders among the most prevalent comorbidities of GD (36, 82, 83) and show substance use itself as a factor consistently associated with GD (41, 64). Furthermore, both factors did not predict GD after one year. Group differences might have been “washed out” for example due to changed substance use during the Covid-19 Pandemic (84) or unknown contextual moderators (cf. 85).

### 4.3 Comorbid mental disorders

As hypothesized, online sports bettors with GD reported more symptoms of the most prevalent comorbid mental disorders besides substance use disorders, namely affective and anxiety disorders (36, 83, 86). Players with GD seemed to experience more signs of depression, anxiety and somatization, which in turn increased the probability for GD after one year by about 13 - 20%. In line with our findings, other studies have found somatoform disorders to be prevalent (17 - 60%) in clinical samples with GD (87–89).

### 4.4 Stress

In support of our hypothesis we found online sports bettors with GD to experience more perceived stress than those without, which is consistent with other studies demonstrating an association of GD with higher perceived stress for example in German adolescents (90). Stress was also relevant in predicting GD one year later as it increases the probability of GD by 17-22%. In addition, perceived helplessness as one of its facets was among the two remaining significant predictors in the longitudinal overall analysis, further supporting the relevance of perceived stress in predicting GD.

In line with our findings there are many studies suggesting that stress plays a role in risky gambling (e.g. 91), e.g. in its onset (e.g. 92), but also in relapse (93). The literature suggests various links (see 93 for an overview) such as a possibly altered stress reactivity in GD similar to substance use disorders, gambling as a stressor, and akin to emotion regulation gambling as a way of coping with stress (e.g. 94).

In sum, these findings emphasize the need to integrate the training of adaptive coping mechanisms like problem solving and stress reduction techniques into GD treatment for online sports bettors. Moreover, these findings reinforce the necessity of routine screenings and/or comprehensive assessment for comorbid mental disorders, suggested before (36, 83).

### 4.5 Limitations

Our study presents several limitations that might restrict the interpretation or generalizability of our results. In terms of representativeness, we obtained our sample from a single provider, implicating a possible selection bias. Even though we asked our sample about other types of gambling and sports betting with other providers to balance this bias, it is not possible to draw conclusions about the exclusive influence of sports betting. Participants did gamble predominantly but not exclusively on online sports betting in our study.

Online gambling and in particular online sports betting in Germany underlies very specific regulations and although online sports betting is legal in a wide range of non-Islamic countries worldwide, legislations concerning legal types of (online) sports betting and respective restrictions for example on live-betting vary widely (see 95 for an overview of studied legislations). Although one might speculate that our results are to some degree transferable to other jurisdictions, there is no detailed data on comparability between different countries yet.

Sociodemographic characteristics in our sample were quite similar to those in other studies (41, 63) and the prevalence of GD in our initial sample (23%) was comparable to other studies combining player tracking data and GD measures (18 - 27%, 27–29), suggesting a representative sample. In addition, we relied on self-report data, which is subject to recall bias.

### 4.6 Conclusion

For the first time, our study used a different, DSM-5 based screening tool in a sample that participates in an increasingly relevant type of gambling, namely online sports betting. To the best of our knowledge, our study was the first to explore the putative individual risk factors examined here in a player tracking data based sample, especially concerning their respective longitudinal relevance in the course of GD. Our cross-sectional results show that the risk profile of individuals with GD in online sports betting is comparable to other gambling activities. For the first time, our study’s longitudinal findings imply the individual risk factors’ relevance over the course of GD in online sports bettors. Explorative analyses suggest that the individual risk factors examined here are especially relevant for predicting stable GD over time. Furthermore, all risk factors seem to be interrelated and share explained variance, suggesting a potential underlying, more general vulnerability. The now more refined risk profile of online sports bettors suggests that established prevention and treatment programs for GD might also be of use. Altogether, our study further stresses the need to individualize GD treatment and respective interventions according to individual risk profiles and needs. For example, regular or comprehensive screenings for comorbid mental disorders in help seeking individuals could aid in establishing such risk profiles. Based on resulting profiles, interventions could comprise the training of specific adaptive coping strategies like problem solving as well as information about the role of impulsivity. The latter should also find its way into prevention efforts. As online sports betting expands, understanding who is at risk of developing problems in respect to this form of gambling will become increasingly important for legislation, prevention and treatment efforts. Assessing further individual risk factors in players of specific gambling types with increasing relevance such as online gambling may support the development of individually tailored treatments in the future. To bridge the gap between etiological and preventive research in the field of online gambling and especially online sports betting, future studies should explore the association between individual risk factors and gambling behavior in the form of player tracking data. The combination of these aspects promises a better understanding and a more complete picture of GD among online sports bettors. A deeper exploration of this association will be of high interest in further investigations and will also be explored in further investigations within the RIGAB study.

## Data availability statement

The datasets presented in this article are not readily available because we received sensitive information like player tracking data. The raw data might be attributed to individuals by the provider, which is why we have to apply further anonymization processes like aggregation before making the data available. This is why we will only be able to make the data available after we have finished the data collection/procession and applied further anonymization procedures to comply with data protection rights and ensure the participants’ anonymity. This will supposedly take place in autumn 2024. Once this is completed, we will make the anonymized data available. Data remaining sensitive will then be available upon reasonable request, i.e. for further research within the used open repository (controlled access) or only in aggregated form. Requests to access the datasets should be directed to theresa.wirkus@tu-dresden.de.

## Ethics statement

The studies involving humans were approved by the Institutional Review Board Ethics Committee of Technische Universität Dresden (IRB00001473) under the reference SR-EK-190032021. The studies were conducted in accordance with the local legislation and institutional requirements. The participants provided their written informed consent to participate in this study.

## Author contributions

TW: Data curation, Formal analysis, Investigation, Project administration, Writing – original draft. RC: Conceptualization, Project administration, Supervision, Writing – review & editing. GB: Funding acquisition, Supervision, Writing – review & editing. AK: Conceptualization, Methodology, Supervision, Writing – review & editing.
