# Supplementary material for: Individual risk factors and prediction of gambling disorder in online sports bettors - the longitudinal RIGAB study
Source: Front Psychiatry. 2024 Feb 27;15:1320592. doi: 10.3389/fpsyt.2024.1320592 (PMC10929711; doi:10.3389/fpsyt.2024.1320592)
Supplement: Supplementary file 1 [file DataSheet_1.docx]

Supplementary Material

# Drop-out analyses

Table S1. Drop-out analyses.

| Outcome | Study retention *Mean (SD)* | Drop-out  *Mean (SD)* | Statistics | *p* |
| --- | --- | --- | --- | --- |
| Age | 35 (8.75) | 33 (8.89) | z = -2.65 | .008 |
| Gender (male) | *n* = 304 (94%) | *n* = 253 (90%) | Χ^2^ = 2.92 | .088 |
| Education |  |  | Χ^2^ = 7.17 | .411 |
| low | *n* = 26 (8%) | *n* = 32 (11%) |  |  |
| middle | *n* = 106 (33%) | *n* = 98 (35%) |  |  |
| high | *n* = 188 (58%) | *n* = 146 (52%) |  |  |
| other | *n* = 5 (2%) | *n* = 6 (2%) |  |  |
| Age of account in years | 5.47 (2.96) | 5.43 (3) | *t*(605) = -.16 | .875 |
| Sum of DSM-5 Stinchfield criteria | 2.22 (2.41) | 1.95 (2.31) | *t*(605) = -1.36 | .175 |
| Substance use |  |  |  |  |
| QFI alcohol | 53.51 (94.5) | 53.75 (107.47) | *t*(605) = .03 | .976 |
| QFI tobacco | 31.62 (52.94) | 40.43 (58.31)  *n* = 280 | *t(603) = 1.95* | .052 |
| Impulsivity |  |  |  |  |
| Sensation seeking | 9.22 (3.49) | 9.2 (3.65) | *t*(605) = -.08 | .938 |
| Lack of premeditation | 8 (2.59) | 8.01 (2.56) | *t*(605) = .05 | .96 |
| Lack of perseverance | 7.33 (2.53) | 7.2 (2.58) | *t*(605) = -.63 | .531 |
| Positive urgency | 7.12 (2.73) | 7.36 (2.97) | *t*(605) = 1.03 | .303 |
| Negative urgency | 8.7 (2.84) | 8.49 (2.98) | *t*(605) = -.86 | .392 |
| Difficulties in emotion identification and Emotion regulation strategies |  |  |  |  |
| Diff. emot. ident. | 12.52 (6.4) | 12.11 (6.13) | *t*(605) = -.81 | .418 |
| Emot. reg. strateg. |  |  |  |  |
| suppression | 3.8 (1.23) | 3.76 (1.28) | *t*(605) = -.37 | .71 |
| reappraisal | 4 (1.11) | 3.9 (1.09) | *t*(605) = -1.19 | .236 |
| Comorbidities |  |  |  |  |
| General Severity Index | 7.24 (9.81) | 6.48 (8.54) | *t*(605) = -1.02 | .308 |
| Depression | 2.86 (4.38) | 2.59 (3.91) | *t*(605) = -.81 | .416 |
| Anxiety | 2.65 (3.5) | 2.23 (2.9) | *t*(605) = -1.6 | .11 |
| Somatization | 1.73 (3.1) | 1.66 (2.93) | *t*(605) = -.29 | .776 |
| Stress |  |  |  |  |
| PSS-10 Sum score | 24.61 (6.32) | 24.58 (5.96) | *t*(605) =-.06 | .956 |
| Perceived helplessness | 14.4 (4.87) | 13.67 (4.79) | *t*(605) = -1.85 | .065 |
| Perceived self-efficacy | 10.21 (2.84) | 10.91 (3.35) | z = 2.2 | .028 |

*Note.* If not otherwise indicated *n_study retention_* = 325, *n_dropout_* = 282. *SD* = Standarddeviation, QFI = quantity-frequency-index, PSS = Perceived Stress Scale.

# Differences in individual risk factors depending on the course of gambling disorder

2.1 Short introduction

In the following, we present explorative results testing whether participants differ in the individual risk factors examined here depending on the course of gambling disorder (GD) over one year. In other words, depending on the possible onset, maintenance or remission of GD.

To examine whether putative risk factors play different roles during the onset, maintenance or remission of GD, we compared the following four groups:

1. GD diagnosis at the time of the initial online survey and one year later (stable GD = maintenance),
2. no GD at the time of the initial online survey, GD at the time of the follow-up (onset),
3. GD at the time of the initial online survey, no GD at the time of the follow-up (remission);
4. no GD at the time of both surveys (stable non-GD).

All risk factors in our study were compared between the reference group 4 (stable non-GD) and the other groups. Corresponding to DSM-5, the clinical cut-off for a diagnosis is four fulfilled criteria. All measures used to assess the individual risk factors are the same as in the original manuscript.

2.2 Statistical analysis

As in the original manuscript, group differences between the four groups were analyzed using robust regressions, separately for each putative risk factor. Dummy-coded group membership was used as the independent variable, with group 4 (stable non-GD) as the reference group. The following individual risk factors examined at the initial online survey of the RIGAB study were each used as outcomes / dependent variables: impulsivity, tobacco and alcohol use, difficulties in emotion identification, emotion regulation strategies, comorbid mental disorders and stress. All analyses controlled for the influence of age, gender, education, the number of days between the surveys and the age of account.

The presented analyses are exploratory and have not been preregistered. The creation of the groups used for analyses is described in the preregistration of the in-person study of the RIGAB study (https://osf.io/g3nfv). The results are partly based on Leopold Schimmank’s master’s thesis.

2.3 Results

There were *n* = 59 (18.15%) participants in group 1 (stable GD). There were *n* = 16 (4.92 %) participants in group 2 (onset). There were *n* = 19 (5.85 %) participants in group 3 (remission) and *n* = 231 (71.08 %) in the reference group 4 (stable no GD). The results of the robust linear regression analyses for differences in putative individual risk factors between different courses of GD can be found in Table S2.

Table S2. Results of the robust linear regression analyses for differences in putative individual risk factors between different courses of gambling disorder.

| Outcome | Regression coefficient | *p* | 95% CI |
| --- | --- | --- | --- |
| **Substance use** |  |  |  |
| QFI alcohol |  |  |  |
| Stable GD | -26.03 | <.001 | [-40.45, -11.6] |
| Onset | 9.41 | .72 | [-42.16, 60.99] |
| Remission | 10.38 | .571 | [-25.63, 46.38] |
| QFI tobacco |  |  |  |
| Stable GD | 11.74 | .175 | [-5.26, 28.73] |
| Onset | 3.08 | .803 | [-21.16, 27.32] |
| Remission | 7.67 | .585 | [-19.97, 35.32] |
| **Impulsivity** |  |  |  |
| Sensation seeking |  |  |  |
| Stable GD | -.47 | .373 | [-1.52, .57] |
| Onset | -.45 | .565 | [-2.01, 1.1] |
| Remission | .78 | .357 | [-.89, 2.46] |
| Lack of premeditation |  |  |  |
| Stable GD | 2.07 | <.001 | [1.34, 2.79] |
| Onset | 1.33 | .005 | [.4, 2.27] |
| Remission | .93 | .182 | [-.44, 2.29] |
| Lack of perseverance |  |  |  |
| Stable GD | 1.95 | <.001 | [1.22, 2.69] |
| Onset | 1.54 | .012 | [.34, 2.74] |
| Remission | 1.08 | .087 | [-.16, 2.33] |
| Positive urgency |  |  |  |
| Stable GD | 1.98 | <.001 | [1.24, 2.72] |
| Onset | 1.03 | .125 | [-.29, 2.34] |
| Remission | 1.78 | .029 | [.18, 3.83] |
| Negative urgency |  |  |  |
| Stable GD | 1.86 | <.001 | [1.08, 2.64] |
| Onset | 1.79 | .001 | [.76, 2.83] |
| Remission | 1.92 | .002 | [.72, 3.12] |
| **Difficulties in emotion identification and emotion regulation strategies** |  |  |  |
| Diff. emot. ident. |  |  |  |
| Stable GD | 5.04 | <.001 | [3.04, 7.04] |
| Onset | 2.2 | .157 | [-.85, 5.26] |
| Remission | 3.86 | .025 | [.5, 7.21] |
| Emot. reg. strateg. |  |  |  |
| Suppression |  |  |  |
| Stable GD | .48 | .007 | [.13, .83] |
| Onset | -.24 | .455 | [-.87, .39] |
| Remission | .07 | .775 | [-.42, .56] |
| Reappraisal |  |  |  |
| Stable GD | .16 | .342 | [-.17, .5] |
| Onset | -.2 | .297 | [-.56, .17] |
| Remission | -.03 | .925 | [-.57, .52] |
| **Comorbidities** |  |  |  |
| General Severity Index |  |  |  |
| Stable GD | 10.17 | <.001 | [6.41, 13.93] |
| Onset | 2.33 | .264 | [-1.77, 6.44] |
| Remission | 6.74 | .052 | [-.06, 13.55] |
| Depression |  |  |  |
| Stable GD | 5.14 | <.001 | [3.5, 6.77] |
| Onset | .87 | .287 | [-.73, 2.47] |
| Remission | 2.61 | .026 | [.32, 4.9] |
| Anxiety |  |  |  |
| Stable GD | 3.19 | <.001 | [1.86, 4.52] |
| Onset | .56 | .543 | [-1.25, 2.37] |
| Remission | 2.12 | .102 | [-.42, 4.65] |
| Somatization |  |  |  |
| Stable GD | 1.84 | .003 | [.62, 3.07] |
| Onset | .91 | .167 | [-.38, 2.2] |
| Remission | 2.02 | .075 | [-.2, 4.24] |
| **Stress** |  |  |  |
| PSS-10 Sum score |  |  |  |
| Stable GD | 7.34 | <.001 | [5.55, 9.13] |
| Onset | 3.42 | .024 | [.44, 6.39] |
| Remission | 5.64 | <.001 | [3.4, 7.87] |
| Perceived helplessness |  |  |  |
| Stable GD | 5.44 | <.001 | [4.01, 6.87] |
| Onset | 3.02 | .015 | [.58, 5.45] |
| Remission | 4.37 | <.001 | [2.36, 6.39] |
| Perceived self-efficacy |  |  |  |
| Stable GD | -1.9 | <.001 | [-2.74, -1.05] |
| Onset | -.4 | .502 | [-1.57, .77] |
| Remission | -1.26 | .07 | [-2.63, .1] |

*Note.* *N* = 325. CI = 95% Confidence Interval, QFI = quantity-frequency-index, GD = gambling disorder, PSS = Perceived Stress Scale. Robust regressions were conducted for each putative individual risk factor separately. The reference group was group 4 (stable no GD), i.e. participants without GD at the time of both surveys. All analyses controlled for the influence of age, gender, education, the number of days between the surveys and age of account.

2.4 Conclusion

The results correspond to the main results in our manuscript. We found the most significant differences concerning the risk factors between the participants with stable GD compared to participants with stable non-GD, for instance concerning alcohol use, impulsivity, difficulties in emotion identification, the use of suppression as emotion regulation strategy, comorbid mental disorders and perceived stress. These findings are in line with cross-sectional evidence, also described in our main manuscript, differentiating between players with and without GD and supporting the stability of these differences over the course of one year.

For the individual risk factors negative urgency, perceived helplessness and the PSS-10 sum score, all groups differed significantly from the group who had no GD at both time-points. All groups displayed on average higher values in these factors than the participants without GD at both time-points. Compared to the stable non-GD group, participants with stable GD displayed the highest values in these putative risk factors, followed by those in remission, followed by those with an onset of GD. Thus these factor seem to be relevant at every stage during the course of GD.

In addition, there were also individual risk factors on which participants who had GD at the initial online survey but not at the follow-up (remission) differed significantly from those who had no GD at both time-points, such as positive urgency, difficulties in emotion identification and depression. Participants in remission displayed on average higher values in these individual risk factors than participants who had no GD at both time-points. The differences in these factors might persist longer. These variables thus seem to be unsuitable for predicting potential improvement in the course of GD, as they differ between healthy gamblers and participants who are not remitted (stable GD) and those in remission.

For lack of premeditation and lack of perseverance we found significant differences between participants who did not have GD at the initial online survey but met the criteria at the follow-up (onset) and participants who had no GD at both timepoints. These results support impulsivity as one of the core mechanisms underlying the development of GD.

While considering the results concerning participants with an onset of GD or in remission, one has to bear in mind that these two groups are rather small (onset *n* = 16, remission *n* = 19) in the current study and conclusions are thus limited. The other two groups, stable GD (*n* = 59 ) and stable non-GD (*n* = 231), are relatively large, which could be one of the reasons why we found the majority of significant differences between the two groups.

Overall, the results of these analyses add longitudinal validity to the individual risk factors’ relevance, especially concerning their ability to differentiate between players with and without GD. The differences in these risk factors seem to persist, some even when players are already in remission. Impulsivity and stress seem to be relevant at every stage during the course of GD, including its onset.
